# The transport function of the human lymphatic system—A systematic review

**DOI:** 10.14814/phy2.15697

**Published:** 2023-06-02

**Authors:** Lene Thorup, Anders Hjortdal, Donna B. Boedtkjer, Morten B. Thomsen, Vibeke Hjortdal

**Affiliations:** ^1^ Department of Cardiothoracic Surgery Copenhagen University Hospital Copenhagen Denmark; ^2^ Department of Clinical Medicine University of Copenhagen Copenhagen Denmark; ^3^ Department of Clinical Medicine Aarhus University Aarhus Denmark; ^4^ Department of Biomedicine, Faculty of Health Aarhus University Aarhus Denmark; ^5^ Department of Biomedical Sciences University of Copenhagen Copenhagen Denmark

**Keywords:** lymphatic function, lymphatic system, lymphedema, physiology, thoracic duct

## Abstract

Physiological properties and function of the lymphatic system is still somewhat of a mystery. We report the current knowledge about human lymphatic vessel contractility and capability of adaptation. A literature search in PubMed identified studies published January 2000–September 2022. Inclusion criteria were studies investigating parameters related to contraction frequency, fluid velocity, and lymphatic pressure in vivo and ex vivo in human lymphatic vessels. The search returned 2885 papers of which 28 met the inclusion criteria. In vivo vessels revealed baseline contraction frequencies between 0.2 ± 0.2 and 1.8 ± 0.1 min^1^, velocities between 0.008 ± 0.002 and 2.3 ± 0.3 cm/s, and pressures between 4.5 (range 0.5–9.2) and 60.3 ± 2.8 mm Hg. Gravitational forces, hyperthermia, and treatment with nifedipine caused increases in contraction frequency. Ex vivo lymphatic vessels displayed contraction frequencies between 1.2 ± 0.1 and 5.5 ± 1.2 min^−1^. Exposure to agents affecting cation and anion channels, adrenoceptors, HCN channels, and changes in diameter‐tension properties all resulted in changes in functional parameters as known from the blood vascular system. We find that the lymphatic system is dynamic and adaptable. Different investigative methods yields alternating results. Systematic approaches, consensus on investigative methods, and larger studies are needed to fully understand lymphatic transport and apply this in a clinical context.

## 1 INTRODUCTION

Since the discovery of human lymphatic tissue dating back to Ancient Greece, our understanding of the lymphatic system has developed significantly (Suy et al., 2016).

Arranged throughout the body much like the venous network, the lymphatic vascular system serves a multitude of functions. Aside from being an essential part of the immune response (Oliver & Alitalo, 2005) it also plays a central role in the uptake of dietary fat. Upon absorption across the intestinal mucosa, fatty acids and monoglycerides are joined together with proteins, cholesterol, and other constituents to form chylomicrons, which are then transported to the bloodstream by the lymphatic vasculature in the form of chyle (Breslin et al., 2018; Cifarelli & Eichmann, 2019; Hokkanen et al., 2019).

Additionally, lymphatic vessels play an important role in transporting interstitial fluid and extravasated proteins back to the systemic circulation thereby maintaining fluid homeostasis (Telinius & Hjortdal, 2019). According to the Starling Principle, tissue fluid homeostasis is determined by the hydrostatic and osmotic pressures in the interstitium and capillaries (Levick, 2004; Woodcock & Michel, 2021). A recent revision of this concept has proposed that due to the endothelial glycocalyx acting as a semipermeable layer between the capillary lumen and the endothelium under steady‐state conditions, the reabsorption into the venous system may not be the major route for returning interstitial fluid to the circulation. As a space is created between the glycocalyx and the endothelium, into which proteins are filtered, it is across this luminal barrier that the osmotic force primarily exerts its effect. However, because of consistent fluid flux from the capillary to the interstitium, these proteins are continuously moved out of this space and into the interstitium, where they do not have any osmotic effect: this essentially obliterates the interstitial osmotic force during steady‐state conditions, which means filtration is driven by the hydrostatic pressure. Since the pressure in the interstitial space will always be negative in steady state, filtration will dominate with practically no absorption into the capillaries (Michel et al., 2020).

Consequently, 8–12 liters of fluid returns to the systemic circulation via the lymphatic vasculature in the course of a day (Scallan et al., 2016). This transport is organized through a vast system of peripheral lymphatic capillaries coalescing into increasingly larger collecting vessels until terminating into the central venous system at the subclavian level (Brotons et al., 2012). The transport is unidirectional, promoted by intraluminal valves and myogenic smooth muscle cells in the lymphatic vessel wall (Adamczyk et al., 2016; Alitalo, 2011), possibly with influence from pacemaker‐like cells located in the lymphatic vessels (Briggs Boedtkjer et al., 2013). Additionally, some of the lymphatic fluid is returned to the blood via lymphatic‐venous anastomoses in lymph nodes. Failure of this system can manifest itself in many forms, from peripheral lymphedema to the centralized organ dysfunction as seen in plastic bronchitis, chylothorax, and protein‐losing enteropathy (Kelly et al., 2020).

Despite this advanced understanding of key processes in the lymphatic system including the origin and destination of interstitial fluid, unanswered questions about the steps along its way remain.

During the last decades, it has become increasingly clear that modifying lymphatic flow is an unmet medical need. The aim of this review is to systematically describe the quantifiable physiological parameters (contraction frequency, velocity, and pressure) of human lymphatic flow function by compiling the knowledge as it is reported in the recent literature.

## 2 METHODS

This review was conducted according to the updated Preferred Reporting Items for Systematic Reviews and Meta‐Analyses (PRISMA) guidelines (Page et al., 2021). A Prisma checklist is available in Appendix S1.

### 2.1 Eligibility criteria

Studies reporting on the physiologic properties of lymphatic vessel function and lymphatic flow parameters in humans or human lymphatic vessels were eligible for inclusion. Physiological parameters were defined as a measurable unit reporting on either: (1) frequency as number of contractions in a given time, (2) velocity reported with quantitative measurements of a distance in a given time, (3) pressure or tension measured in any relevant unit. Only studies reporting original data in humans and from human tissue were considered.

The following exclusion criteria were used for in vivo studies: (1) data presented without measurable parameters, for example, transit time without transit distance, (2) reporting of data from diseased lymphatic vessels without the inclusion of a control group, either healthy controls or acting as own control in a nonaffected limb, (3) not written in English, (4) nonhuman participants, (5) the article was a review, letter, comment, meta‐analysis, or conference abstract, (6) articles published before January 1st, 2000. The same exclusion criteria were used for ex vivo studies except from the inclusion of lymphatic vessels harvested from patients.

### 2.2 Search strategy

A systematic search was carried out in PubMed, using a combination of both MeSH terms and Title/Abstract related to “lymphatic function”, “physiology”, and “pharmacology”. A detailed search string is available in the Appendix S1. The search was carried out in April 2022 and updated in September 2022. Reference lists of included studies were screened to obtain additional literature. A structured reference review of the included studies was conducted to confirm all relevant articles were included.

### 2.3 Study selection and data extraction process

This review utilized Covidence.org (Covidence systematic review software (2022) for reference management. After the removal of duplicates, authors AH, VH, and LT independently screened the articles by title and abstract for relevance. Eligible studies were then independently assessed by the same three authors, referring to the previously defined inclusion and exclusion criteria. At each step, at least two reviewers had to agree on either exclusion or inclusion. Any disagreement was solved by consensus in the reviewer group.

Studies were sorted into either in vivo or ex vivo designs. Data extracted from in vivo studies included baseline characteristics: author, year of publication, number of participants, age, comorbidities, functional parameters (lymphatic flow velocity and/or pressure; frequency of vessel contraction), study intervention (if any), control group (if any), and postintervention functional parameters. Ex vivo data included the same basic information along with contraction frequency and two measures of tension properties: passive/baseline and active. Passive tension is the tension measured in the isolated vessel at its relaxed, baseline level. Active tension is the tension measured at spontaneous or stimulated contraction minus the baseline tension.

Figures were created using GraphPad Prism (version 8.0.2 for Windows, GraphPad Software, San Diego, California USA), displaying the distribution of functional parameters in in vivo studies, reported with mean and standard deviation when available. A comparison of the effect of interventions was also plotted. Units for each parameter were defined as (1) frequency: vessel contractions per minute (2) fluid pressure: mm Hg, (3) fluid velocity: centimeters per second. Any parameters stated in other units in the original articles were converted to these predefined units for the plots and tables.

## 3 RESULTS

**FIGURE 1:**
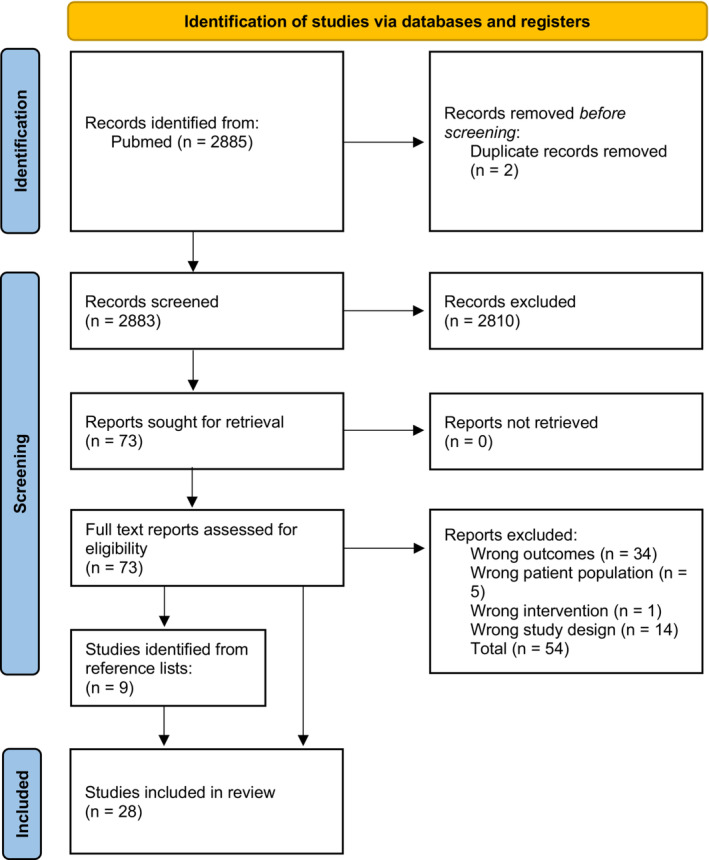
PRISMA 2020 flow diagram of inclusion of papers for systematic review.

The PubMed search returned 2885 results. Title and abstract screening excluded 2810 papers and of the remaining 73 papers, 21 were found eligible upon full‐text screening. Screening of reference lists of included papers yielded an additional seven articles for inclusion. In total, 28 papers (Amann‐Vesti et al., 2003; Belgrado et al., 2016; Bell et al., 2020; Gray et al., 2016; Groenlund et al., 2017; Holm‐Weber et al., 2022; Kelly, Mohanakumar, Telinius, et al., 2020; Lopera et al., 2017; Majgaard et al., 2022; Modi et al., 2007; Mohanakumar et al., 2018, 2019, 2020, 2021; Rane et al., 2013; Rasmussen et al., 2010, 2021, 2022; Saito et al., 2015; Tan et al., 2011; Telinius et al., 2010, 2015, 2017; Telinius, Baandrup, et al., 2014; Telinius, Kim, et al., 2014; Telinius, Mohanakumar, et al., 2014; Unno et al., 2010; Yamamoto et al., 2014) met the inclusion criteria, see Figure 1.

Of these, 20 were in vivo studies, seven were ex vivo and one study contained both in vivo and ex vivo data. Studies were published between 2003 and 2022, with the majority from 2010 and up. The method most often used to investigate the lymphatic parameters in vivo was near‐infrared fluorescence imaging (NIRF) using indocyanine green (ICG) as the contrast agent (86%). The participants examined included both healthy people and patients with a Fontan circulation, lymphedema, breast cancer, Fabry's disease, venous insufficiency, lipedema, and rheumatoid arthritis. In vivo study characteristics and lymphatic functional parameters are summarized in Table 1.

**TABLE 1:** Summary of in vivo functional properties.

In Vivo Summary																			
Reference	*N*	Age, Years	Control group	Morbidity	Method	Limb	Baseline						Intervention						
							Control/healthy			Diseased			Type of intervention	Healthy			Diseased		
							Frequency (contractions/min)	Pressure (mm Hg)	Velocity (cm/s)	Frequency (contractions/min)	Pressure (mm Hg)	Velocity (cm/s)		Frequency (contractions/min)	Pressure (mm Hg)	Velocity (cm/s)	Frequency (contractions/min)	Pressure (mm Hg)	Velocity (cm/s)
Amann‐Vesti (2003) Lymphatic Research and Biology	22	15–62	Healthy	Fabry Disease	Lymphatic capillary puncture	Leg		6.9 (1.5‐13)			LE: 13.6 (7.8–17.5) No LE: 4.5 (0.5–9.2)								
Belgrado (2016) Lymphatic Research and Biology	30	43 ± 14	NA	Healthy	NIRF (ICG)	Arm							Flush and fill		86 CI ± 3.7 (Occlusion pressure)				
Bell (2020) Arthritis Rheumatol.	13	49 ± 14.3	Healthy	Rheumatoid arthritis	NIRF (ICG)	Hand	0.51 ± 0.35			0.53 ± 0.39									
Gray (2016) Medical Engineering and Physics	10	24–61	NA	NA	NIRF (ICG)	Arm			Median 0.8 (0.4–2)^a^				Mechanical loading (60 mm Hg)			Median 0.6 (0.2–1.4)^a^			
Groenlund (2017) Lymphatic Research and Biology	10	20–30	NA	Healthy	NIRF (ICG)	Leg	0.59 ± 0.13	56 ± 9–57 ± 9	1.51 ± 0.24				Hyperthermia Exercise 10‐min postexercise	1.46 ± 0.5 0.68 ± 0.25 0.35 ± 0.19		1.83 ± 0.64 2.2 ± 0.63 1.83 ± 0.64			
Holm‐Weber (2022) Physiological Reports	17	Male: 29 ± 2.7 Female: 27 ± 2.6	NA	Healthy	NIRF (ICG)	Leg	0.5 ± 0.2		1.50 ± 0.4				Increased gravitational force	1.2 ± 0.5		1.7 ± 0.42			
Kelly (2020) Lymphatic Research and Biology	10	25.7 ± 1.3	NA	Healthy	NIRF (ICG)	Arm	0.9 ± 0.4	59 ± 12	1.1 ± 0.3				Handgrip exercise Hyperthermia	0.9 ± 0.4 1.5 ± 0.5		1.2 ± 0.3 1.1 ± 0.4			
Lopera (2017) Lymphatic Research and Biology	9	36 (22–58)	NA	Healthy	NIRF (ICG)	Arm	Forearm: 0.8^a^ Elbow: 1.4^a^		Forearm: 0.76^a^ Elbow: 1.08^a^				Manual lymph drainage Compression garment	Forearm: 1^a^ Elbow: 1.4^a^ Forearm: 1.2^a^ Elbow: 1.8^a^		Forearm: 1.33^a^ Elbow: 1.19^a^ Forearm: 1.0^a^ Elbow: 1.14^a^			
Modi (2007) J Physiol	39	Uncuffed: 52 ± 10 Cuffed: 54 ± 6 BCRL: 60 ± 8	Healthy	BCRL	99mTc‐HIG dermal injection	Arm		39 ± 14	Uncuffed: 0.15 ± 0.1^a^ Cuffed: 0.13 ± 0.18^a^		24 ± 19	0.05 ± 0.15^a^							
Mohanakumar (2019) Circ Cardiovasc Imaging	20	Fontan: 24 ± 7 Control: 26 ± 2	Healthy	Fontan circulation	NIRF (ICG)	Leg	0.5 ± 0.1	60.3 ± 2.8	1.9 ± 0.2^a^	0.8 ± 0.1	50.8 ± 3.1	2.3 ± 0.3^a^							
Mohanakumar (2020) Lymphatic Research and Biology	16	56.5 ± 6.9	NA	Healthy	NIRF (ICG)	Leg	0.4 ± 0.3	54.7 ± 9.4	1.5 ± 0.7				Amlodipine	0.4 ± 0.2	53.9 ± 13.9	1.8 ± 1.0			
Mohanakumar (2021) Physiological Reports	48	Fontan: 27 ± 7 Control: 27 ± 9	Healthy	Fontan Circulation	NIRF (ICG)	Leg	0.3 ± 0.2	57.2 ± 8.4	1.6 ± 0.5^a^	0.4 ± 0.3	54.8 ± 16.2	1.8 ± 0.8^a^	Hyperthermia (5min)	1.4 ± 1.0		Unchanged	0.9 ± 0.5		0.5 ± 0.7
Rane (2013) Radiology	12	31–64	Healthy	Mastectomy w/LE	Spin Label Measurement	Arm			0.01 ± 0.002^a^			0.008 ± 0.003^a^							
Rasmussen (2010) Translational Oncology	44	Healthy: 38.2 ± 11 Diseased: 49.7 ± 17.6	Healthy and Own Control	LE	NIRF (ICG)	Arm and Leg	H‐Arm: 1.3 ± 1.2 OC‐Arm: 1.2 ± 1.0 H‐Leg: 0.4 ± 0.3 OC‐Leg: 0.3 ± 0.2		H‐Arm: 0.8 ± 0.4 OC‐Arm: 0.8 ± 0.4 H‐Leg: 0.9 ± 0.7 OC‐Leg: 0.8 ± 0.5	Arm: 0.3 ± 0.3 Leg: 0.2 ± 0.2		Arm: 0.7 ± 1.0 Leg: 0.8 ± 0.4							
Rasmussen (2021) J Vasc Surg Venous Lymphat Disord	20	CVI: 53.5 (38–70) Control: 43.0 (30–58)	Healthy	Chronic venous insufficiency	NIRF (ICG)	Leg	0.9 ± 0.4			C2: 0.9 ± 0.2 C3: 1.1 ± 0.6 C4: 0.6 ± 0.4									
Rasmussen (2022) Obesity	29	23–58	Healthy	Lipedema	NIRF (ICG)	Leg	0.9 ± 0.4			Lipedema Stage: Stage 1: 1.4 ± 0.6 Stage 2: 1.4 ± 0.7 Stage 3: 1.8 ± 0.1									
Saito (2015) Lymphatic Research and Biology	465	30–85	NA	Healthy	NIRF (ICG)	Leg		30–39 years: 26.9 ± 16.2 40–49 years: 24.7 ± 15.5 50–59 years: 22.3 ± 15.1 60–69 years: 20.6 ± 14.6 70 years: 19.5 ± 14.4											
Tan (2011) Arch Phys Med Rehabil	22	18–68	Healthy and Own Control	LE	NIRF (ICG)	Arm and Leg			H‐Arm: 0.7 ± 0.32 H‐Leg: 0.94 ± 0.80 A‐Arm: 0.68 ± 0.29 A‐Leg: 0.71 ± 0.35			Arm: 1.04 ± 0.81 Leg: 0.62 ± 0.32	Manual lymph drainage			H‐Arm: 0.80 ± 0.40 H‐Leg: 1.27 ± 1.11 A‐Arm: 0.87 ± 0.34 A‐Leg: 0.76±0.59			Arm: 1.19 ± 0.69 Leg: 0.95 ± 0.66
Telinius (2014) J Physiol	6	26 ± 0.4	Healthy (Placebo)	Healthy	NIRF (ICG)	Leg	0.77 ± 0.15	58 ± 3.8	1.2 ± 0.2^a^				Nifedipine	1.05 ± 0.16	58 ± 3.1	1.3 ± 0.2^a^			
Unno (2010) J Vasc Surg	65	58.5 ± 13.5 71.9 ± 12.1	Healthy and AAA	LE	NIRF (ICG) & DynLS	Leg		Sitting NIRF: 29.3 ± 16.0 Supine NIRF: 25.2 ± 16.7 Supine DynLS: 26.4 ± 16.5			Sitting NIRF: 13.2 ± 14.9								
Yamamoto (2014) Ann Plast Surg	15	41‐74	Own control	Breast cancer w/unilateral LE	NIRF (ICG)	Arm			0.5 ± 0.3^a^			ISL 0: 0.2 ± 0.2^a^ ISL 1: 0.09 ± 0.1^a^ ISL 2: 0.03 ± 0.02^a^ ISL 3: 0.01 ± 0.004^a^ ADB 1: 0.35 ± 0.0^a^ ADB 2: 0.13 ± 0.11^a^ ADB 3: 0.04 ± 0.01^a^ ADB 4: 0.01 ± 0.005^a^ ADB 5: 0.008 ± 0.002^a^							

*Note*: () indicates range. Values listed as mean ± standard deviation unless otherwise indicated.

Abbreviations: AAA, abdominal aortic aneurism; A‐Arm, asymptomatic arm; ADB, arm dermal backflow stage; A‐Leg, asymptomatic leg; BCRL, breast cancer treatment‐related lymphedema; C2‐C4, clinical, etiological, anatomical, and pathophysiological (CEAP) classification system of venous insufficiency stage 2‐4; CVI, chronic venous insufficiency; DynLS, dynamic lymphoscintigraphy; H‐Arm, healthy arm; H‐Leg, healthy leg; ICG, Indocyanine Green; ISL, International Society of Lymphology stage; LE, lymphedema; NIRF, near‐infrared flourescence imaging; OC‐Arm, own control arm; OC‐Leg, own control leg; SD, standard deviation.

^a^ Converted from original values.

### 3.1 In vivo results

#### 3.1.1 Baseline functional properties of peripheral lymphatic vessels in healthy individuals

**FIGURE 2:**
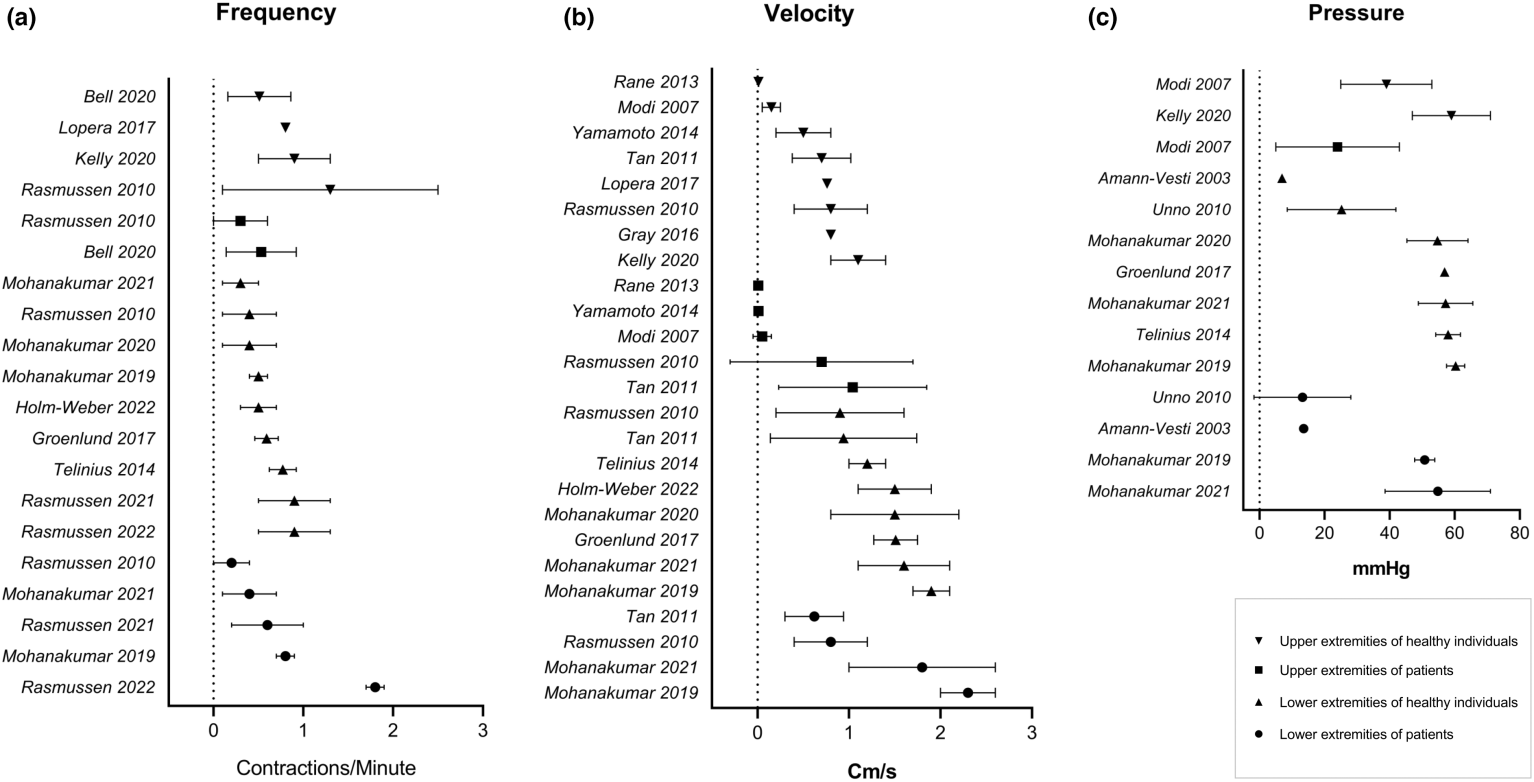
Plot of baseline values of lymphatic vessel (a) contraction frequency; (b) velocity of lymphatic fluid; (c) lymphatic pressure in the upper and lower extremities from both healthy participants and patients. Values reported as mean ± SD where available.

The contraction frequency of relaxed lymphatic vessels from healthy participants ranged from 0.5 to 1.3/min in the upper extremities and 0.3 to 0.9 min^−1^ in the lower extremities (Figure 2a).

Lymphatic pressure was between 39 ± 14 and 59 ± 12 mm Hg in the upper extremities and between 6.9 and 60.3 ± 2.8 mm Hg in the lower extremities (Figure 2b). The lower measurement of 6.9 (range 1.5 to 13) mm Hg was assessed using lymphatic capillary puncture as the only study utilizing this method (Amann‐Vesti et al., 2003). If limiting to only including NIRF imaging, the pressure range in the lower extremities was between 19.5 ± 14.4 and 60.3 ± 2.8 mm Hg.

Finally, the velocity of lymphatic movement in the upper extremities ranged from 0.01 ± 0.002 to 1.1 ± 0.3 cm/s (Figure 2c). The lower limit was measured using spin labeling magnetic resonance imaging method as the only study (Rane et al., 2013). If using NIRF imaging only, the range was 0.76 to 1.1 ± 0.3 cm/s. In the lower extremities this range was 0.9 ± 0.7 to 1.9 ± 0.2 cm/s.

Generally, the velocity appeared slightly higher in the lower extremities compared with the upper, and the contraction frequency was the opposite—slightly lower in the lower extremities. The lymphatic pressure did not seem to differ much between extremities.

#### 3.1.2 Baseline functional properties of peripheral lymphatic vessels in patients

Contraction frequency did not differ significantly with disease except one study which found lower frequencies in the upper and lower extremities affected by lymphedema (Rasmussen et al., 2010) and another study which on the contrary found an increase in contraction frequency of lower extremities affected by lipedema (Rasmussen et al., 2022) (Figure 2a).

Pressure in investigated lower extremities of Fontan patients and upper extremities of breast cancer‐related lymphedema was generally lower compared to healthy controls (Modi et al., 2007; Mohanakumar et al., 2019, 2021). One study did, however, find a higher pressure in the legs of Fabry patients with lymphedema (Amann‐Vesti et al., 2003) (Figure 2b).

The lymph velocity was generally lower in the upper extremities affected by lymphedema except in one study which showed an increase (Tan et al., 2011) (Figure 2c). One study demonstrated a linear relationship between lower velocity and progressing disease stage (Yamamoto et al., 2014). Velocity in the lower extremities remained either unchanged, increased or decreased in various diseases, and thus no clear, unifying tendency was found.

#### 3.1.3 Effect of interventions

**FIGURE 3:**
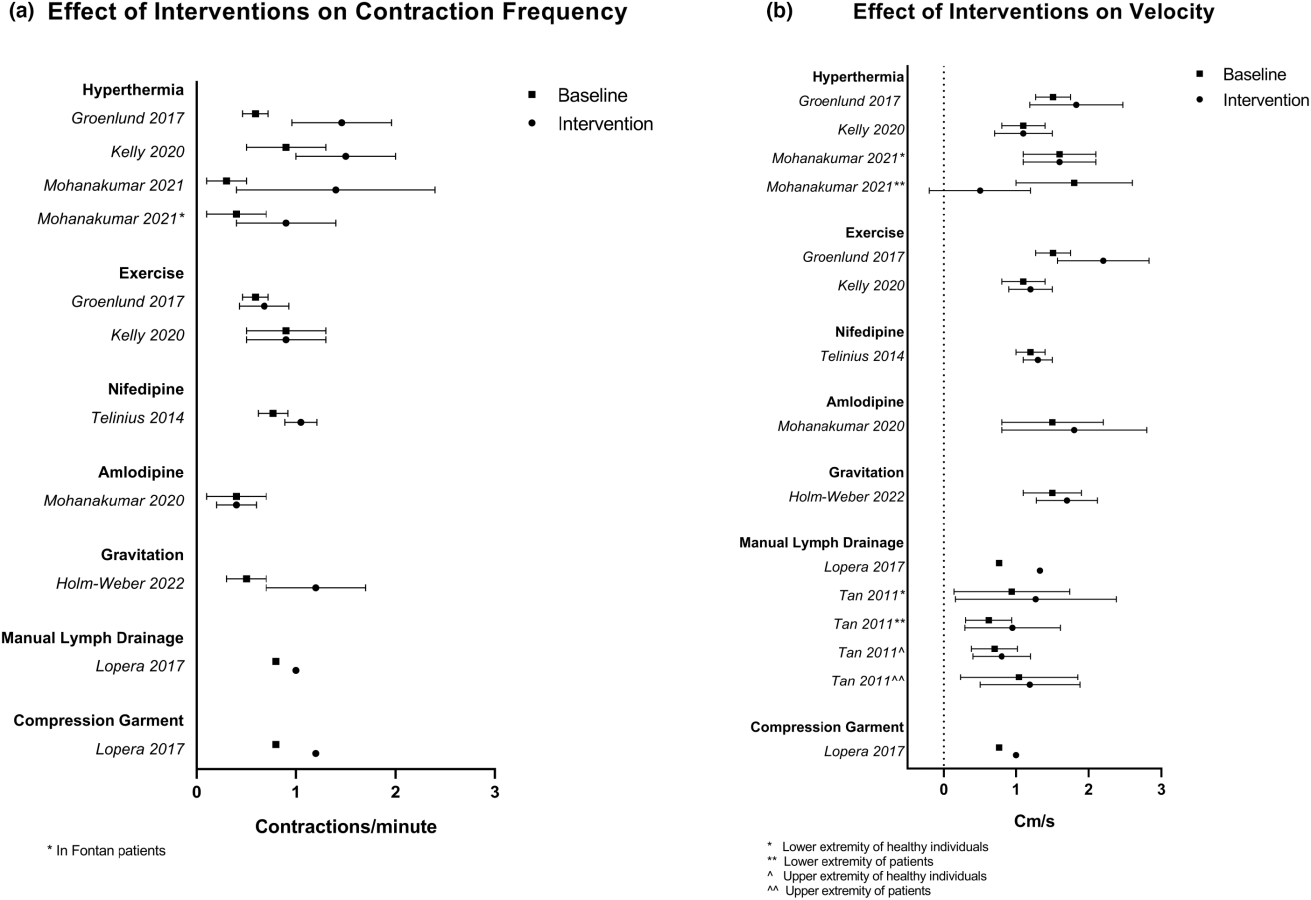
Plot of baseline and postintervention values of (a) contraction frequency and (b) velocity. All values reported as mean and where available ± SD.

Exposure of limbs to various stimuli resulted in changes in contractile function. Hyperthermia, exposure to increased gravitational forces, and treatment with nifedipine all caused an increase in contraction frequency. This was not the case for pressure or velocity, where a tendency toward an increase is noted, but some studies also found unchanged values (Figure 3).

### 3.2 Ex vivo results

All ex vivo studies were carried out in lymphatic vessels obtained from diseased patients. Two types of vessels have been examined: the thoracic duct (TD) and mesenteric lymphatic vessels (MLV). All studies reported here utilized a wire myograph setup for examinations. Contraction frequencies preactivation ranged between 2.1 ± 0.7 and 5.5 ± 1.2 min^−1^ in MLVs and 1.2 ± 0.1 and 4.3 ± 0.4 min^−1^ in TDs. Noticeably, some vessels only exhibited spontaneous contractions after physical stimulation (occurring during the exchange of physiological solution in myograph) or upon vasoconstriction with agonists and subsequent washout. These postactivation contraction frequencies were within the same range as preactivation (or spontaneous) frequencies, except for ivabradine which revealed a markedly higher postactivation frequency. Ex vivo parameters are summarized in Table 2.

**TABLE 2:** Summary of ex vivo functional properties.

Ex vivo summary													
Reference	*N* ^a^	Age, Years	Type of vessel	Avg, diameter^b^ (mean ± SD)	Morbidity	Baseline		Intervention					
						Frequency^c^ (contractions/minute)	Baseline tension (Nm‐1)	Type of intervention	Concentration	Overall effect of intervention	Frequency change (contractions/minute)	Passive tension (Nm‐1)	Avg. active tension (Nm‐1)
Majgaard (2022) Physiological Reports	66	Range 40–84	TD MLV	1675 ± 97 μm (±SEM) 346 ± 36 μm (±SEM)	Esophageal and cardia cancer GBP	4.3 ± 0.4 4.4 ± 0.5		Ivabradine ZD7288 Cesium Pyrilamine Histamine	≥10 μm ≥30 μm 5 mM 10 μM 0.1 pM—10 μM	Contraction Contraction No change No effect No effect	↑ ↑ ↑ – –		
Mohanakumar (2018) Am J Physiol Heart Circ Physiol	42	64.8 ± 1.6 (±SEM) 64.8 ± 1.6 (±SEM)	TD MLV	1.47 mm 340 μm	Esophageal and cardia cancer GBP/IPAA	2.9 ± 0.6 5.5 ± 1.2		Extracellular Cl^−^ substitution NE CCRC (Cl^−^ subst) DIDS Furosemide DIDS + Furosemide NPPB CPA	200 μM 20 μM 10 nM—100 μM 10 μM	Inhibition of NE contraction Inhibition of NE contraction No change to NE contraction Inhibition of NE contraction Inhibition of spontaneous contraction Contraction	↓/stop ↓/stop ↓/stop ↓/stop ↓/stop ↑		
Telinius (2010) Am J Physiol Heart Circ Physiol	26	64 ± 12 (±SD)	TD	2,21 (95% CI 1.1–3.35)	Esophageal and cardia cancer			Length‐tension NE ET‐1 U46619 ACh Bradykinin L‐NAME L‐NAME + indomethacin	1 nM—10 μM 1 pM—0.1 μM 1 pM—0.1 μM 10 μM 1 μM 100 μM 100 μM + 1 μM	Contraction Contraction Contraction Relaxation (of precontracted) Relaxation (of precontracted) Contraction Contraction	↑ ↑ ↑ ↓ ↓ ↑ ↑	3.11 ± 0.67	6.24 ± 0.75 (max L‐T) 2.59 ± 0.36 5.31 ± 1.3 5.62 ± 1.2
Telinius (2014) J Physiol	65	Range 33–85	TD MLV	≈2 mm ≈300 μm	Esophageal and cardia cancer GBP	1.2 ± 0.1 2–3/min		Nifedipine Verapamil NE + Nifedipine	0.1 nM—1 μM 0.1 nM—3 μM 10 nM—10 μM/20 nM	Inhibition of phasic activity Inhibition of phasic activity Reduction of maximum response to NE	↓ ↓ –		
Telinius (2014) Am J Physiol Heart Circ Physiol	54	65 ± 1 (±SEM)	TD MLV	Not stated Not stated	Esophageal and cardia cancer GBP	2.2 ± 0.3 2.1 ± 0.7	0.56 ± 0.16	TEA Barium Paxilline Apamin TRAM‐34 Apamin + TRAM‐34 NS309 4‐AP Glibenclamide Pinacidil K extracellular Cromakalim	1 mM 30 μM 40 μM + 1 mM 10 mM	Contraction Contraction No change in tone No effect No effect No effect No effect Tansient increase (few mins)	↑ ↑ ↑ – – – – ↑ (transient) ↑ ↓/stop ↑ ↓/stop	0.31 ± 0.1	
Telinius (2014) Am J Physiol Heart Circ Physiol	35	63 ± 10 (±SD)	TD		Esophageal and cardia cancer			NE NPY SP Acetycholine or MCh ACh (or MCh) + atropine Tyramine Electric field stimulation	10 μM 1 μM 10 μM 10 μM 10 μM 2—20 Hz	Contraction (variable) Contraction (variable) Contraction (variable) Contraction (variable) No contraction Contraction Contraction	(Apparent increase in freq) ↑		
Telinius (2015) J Physiol	62	Range 30–82	TD MLV	Not stated Not stated	Esophageal and cardia cancer GBP			TTX Veratridine		Inhibition of phasic activity Contraction	↓/stop ↑	Gain of tone in both TD and MLV	
Telenius (2017) Lymphatic Research and Biology	44	Range 19–69	TD MLV MLV	Not stated Not stated Not stated	Esophageal and cardia cancer GBP IPAA	3.4 ± 1.7 Range ≈1–6/min		SP (TD) SP (MLV; GBP) NE (MLV; IPAA)	0.1 nM—10 μM 0.1 nM—10 μM 1 nM—10 μM	Little or no effect Vasorelaxation (lower BL tone) Contraction	Not analysed Not analysed ↑	0.64 ± 0.46 0.47 ± 0.24	0.93 ± 0.45 0.78 ± 0.4

Abbreviations: 4‐AP, 4‐aminopridine; ACh, acetylcholine; CCRC, cumulative concentration‐response curve; CPA, cyclopiazonic acid; ET‐1, endothelin‐1; GBP, gastric bypass; IPAA, ileal pouch‐anal anstomosis; L‐NAME, *N* ^*G*^‐nitro‐L‐arginine methyl ester; MCh, metacholine; MLV, mesenteric lymphatic vessel; NE, norepinephrine; NPPB, 5‐nitro‐2‐(3‐phenylpropylamino) acid; NPY, neuropeptide Y; SD, standard deviation; SP, substance P; TD, thoracic duct; TEA, tetraethylammonium; TTX, tetrodotoxin.

^a^ Number of patients is how the *N* values are defined in all papers (the number of segments can be much higher in each experiment, but the patient average is reported if there has been a repeated experiment).

^b^ Diameter, as derived from the internal circumference in the passive length‐tension normalization (at 21 mm Hg for TD and 22 mm Hg for MLV).

^c^ Frequency, either spont (after normalization but before application of noradreneline) or with‐ or after‐stimulation (has been given small amount of noradrenaline, or has become “spontaneous” after exposure to agonist and washout).

Several pharmacologic agents have been examined with the lymphatic vessels showing reactivity when exposed to drugs that directly and indirectly affect cation and anion channels of vascular smooth muscle cells, adrenoceptors, and changes in length–diameter relationship (stretching of the lymphatic vessel).

Generally, lymphatic vessel reactivity to known vasoconstrictors and dilators corresponds well to what is described in the blood vascular system. That is, vasoconstrictors such as norepinephrine and endothelin‐1 induced increased contraction frequencies and tone in the lymphatic vessels, while vasodilators such as bradykinin and acetylcholine typically induced relaxation. Ca^2+^, Na^+^, and Cl^−^ channel antagonists along with K^+^ channel agonists all decrease or complete stop of contractions, while K^+^ channel antagonists and Na^+^ agonist increase contraction frequency.

Despite evidence of HCN/funny channels existing in lymphatic vessels, exposure to HCN/funny channel antagonists, known to lower frequency in cardiac pacemaker cells, increased contraction frequency in the lymphatic vessels. This positive chronotropic effect was only evident at supraoptimal concentrations, and unlikely a direct effect via HCN channels.

## 4 DISCUSSION

This systematic review identified 28 papers about human lymphatic vascular functional properties in vivo and ex vivo over a span of 22 years.

The overall impression is that of a dynamic system with the ability to adapt to various stimuli and demands. This adaptation can occur within minutes as shown in the cases of physical stress with hyperthermia, manual compression, and changes in gravitational influence. All stimuli investigated induced either an increase or unchanged lymphatic frequency and velocity in healthy arms and legs in vivo.

While correlating greater frequency to greater flow seems appealing, it is important to remember that contraction force also plays a significant role—similar to stroke volume in cardiac output (Vincent, 2008). In vivo lymphatic contraction force is often approximated by pumping pressure, but with the current methods this only takes into account the superficial vessel pressures. The actual amount of fluid moved in the limb is challenging to determine, due to both the small volume and acellular nature of lymph fluid, which makes noninvasive techniques such as ultrasound suboptimal. Thus, a higher frequency does not necessarily equal a higher flow or more “effective” lymphatic function. To prove or dismiss that assumption, studies that examine the in vivo relationship between the various parameters as well as considering the whole lymphatic system are needed.

Results from ex vivo studies are not directly translatable to in vivo effects, as demonstrated in the study by Telinius, Mohanakumar, et al. (2014). Here, the calcium channel blocker nifedipine decreased contraction frequency ex vivo whereas in vivo testing resulted in an increase in frequency. The mechanism behind this difference is unclear, but the ex vivo study focused on central lymphatic vessels such as the mesenteric and thoracic duct, whereas the in vivo study targeted the peripheral vessels. There could be differences in channels and receptors in the central versus peripheral lymphatic vessels. Testing this would require the donation of peripheral tissue and techniques to dissect and mount these.

Aside from characterizing basic lymphatic physiology, many studies have tended to focus on ways to stimulate and increase lymphatic function. This emphasizes the need for treatment options for the dysfunctional lymphatic circulation as seen in lymphedema, or recently as proposed and described in an animal heart failure model (Abraham et al., 2021). Another example is the inhibitory effects of acidosis on lymphatic contraction frequency (Moeller et al., 2019), making the lymphatic system a potential target in the treatment of acidosis‐associated edema. However, it is also worth highlighting some of the potential advantages that come with lowering the movement of lymphatic fluid. This could be applicable in, for example, snakebite patients where the lymphatic system plays a key transport role. Animal studies of snake bites have suggested a significant delay in time to mortality after topical application of nifedipine (van Helden et al., 2014). This topical administration route has not been tested in humans, but it does correspond well with the ex vivo findings from Telinius, Mohanakumar, et al. (2014).

The relatively small inclusion number in this review does not reflect lack of studies investigating the lymphatic vascular system per se, but most of these studies utilize methods that examine the morphological features of the lymphatics rather than functional (Munn & Padera, 2014; Polomska & Proulx, 2021). While this underlines the novelty of this new field—as well as the opportunities—it is also a work in progress, and with that comes a number of limitations related to especially study design and methods. Here we list some of the most obvious and important limitations encountered.

First, there is a general issue with a small sample size reducing the power of the studies comparing groups of patients or measuring the effect of an intervention. One study (Saito et al., 2015) did include a larger study population of 465 participants and was able to show an inverse relationship between age and lymphatic pressure. Others present data including few participants in each group, the smallest study including only six participants in total.

Secondly, the investigative and analytical methods are characterized by subjective interpretation, resulting in uncertainty of measurements and low external validity of each study. This is also evident in some of the relatively large SDs reported. Some studies have tried to address the analytical variation by doing multiple, independent analyses for each data set. These studies report high intraclass correlation coefficients as an expression of low interobserver variation. The parameter displaying the highest variation was the velocity measurements (Kelly, Mohanakumar, Telinius, et al., 2020). This could be an argument for creating more standardized methods for assessing this parameter in particular.

Finally, comparing studies utilizing different investigative methods poses great difficulties. The results change dramatically when the lymphatic function is studied by lymphatic capillary puncture or spin label measurement compared to NIRF investigations. Most of the identified studies use the NIRF method for investigation, and although their measurements appear to be somewhat consistent, it is unclear whether this is due to the method's superiority or simply its widespread adoption. It is also relevant to consider the lack of investigations of the deeper lymphatic system in these studies. The NIRF imaging system has a maximum depth range of 1–2 cm, thus the estimation of the overall function and transport capacity of the lymphatic system as a whole cannot be assessed using these techniques. Interestingly, the one study utilizing spin labeling MRI to visualize deeper lymphatic vessels (Rane et al., 2013) found dramatically lower flow velocities in both healthy and lymphedema‐affected upper extremities compared to NIRF and lymphoscintigraphy results. Perhaps a sign that deeper lymphatic vessels operate differently than the superficial. However, a literature search yielded no other studies using the same spin label technique, making it difficult to exclude the possibility of it simply being a case of conflicting methods. One other study using another MRI technique (contrast‐enhanced) was identified (Borri et al., 2015). It included three patients and displayed a velocity of 0.035 cm/s in the upper extremities affected by breast cancer‐related lymphedema and 0.16 cm/s in the ipsilateral, nonaffected arm. Thus, a higher velocity than measured using spin labeling MRI (0.008 to 0.01 cm/s) but still drastically lower compared to the NIRF investigations. In conclusion, these two MRI studies measuring the function of deeper lymphatic vessels indicate lower velocity scores compared to superficial vessels, but it is hard to make a direct comparison due to different methods and a very small sample size (six and three participants).

## 5 CONCLUSION

Despite the potential benefits of a more profound knowledge about lymphatic transport function, interest in this area still seems modest. Both in vivo and ex vivo human studies gives the impression of a vascular system that is highly adaptable to physiological as well as pharmacological stimuli. This creates potential for new target areas in the treatment of lymph‐related diseases ranging from heart failure to neglected tropical diseases such as snakebite envenoming. So far, investigations of lymphatic transport function have shown promising results, but there is still a long way to go before applying it in a clinical context. It is clear, that more systematic approaches are needed if we are to fully understand this underappreciated and complex vascular system and eventually be able to treat patients with new lymphatic‐specific treatments.

## FUNDING INFORMATION

During the writing of this review, authors LT and VH were partly funded by Bent Thorberg's Foundation (private foundation) and a common grant from The Novo Nordisk Foundation (grant #NFF17SA0030576), respectively.

## ETHICS STATEMENT

This review is not subject to ethical approval, as only previously published data is reported.

## CONFLICT OF INTEREST STATEMENT

The authors declare no financial conflicts of interest.

## Supporting information


Appendix S1
