# Supplementary material for: The transport function of the human lymphatic system—A systematic review
Source: Physiol Rep. 2023 Jun 2;11(11):e15697. doi: 10.14814/phy2.15697 (PMC10238785; doi:10.14814/phy2.15697)
Supplement: Supplementary file 1 — Appendix S1 [file PHY2-11-e15697-s001.zip › PHY2_15697_PHYSREP-2023-03-019-s03.docx]

Search string for:

The Transport Function of the Human Lymphatic System – A Systematic Review

(("Lymphatic System"[Mesh]) AND (("Physiology"[Mesh]) OR ("Pharmacology"[Mesh]))) OR ((lymphatic[Title/Abstract]) AND (((physiol*[Title/Abstract]) OR (pharmacol*[Title/Abstract])) OR (function[Title/Abstract]))) AND ((humans[Filter]) AND (2000/1/1:2022/4/20[pdat]) AND (english[Filter]))
