# Supplementary material for: The transport function of the human lymphatic system—A systematic review
Source: Physiol Rep. 2023 Jun 2;11(11):e15697. doi: 10.14814/phy2.15697 (PMC10238785; doi:10.14814/phy2.15697)
Supplement: Supplementary file 1 — Appendix S1 [file PHY2-11-e15697-s001.zip › PHY2_15697_PHYSREP-2023-03-019-s01.docx]

Josephine C. Adams

Editor-in-Chief, Physiological Reports

University of Bristol, UK

March 12, 2023

Re: The Transport Function of the Human Lymphatic System – A Systematic Review

Dear Dr. Adams,

We hereby submit this systematic review on the physiological properties and transport function

of the human lymphatic system.The review is written by invitation from previous editor Tom Kleiman and associate editor dr Morten Bækgaard Thomsen.

The systematic review summarises the current knowledge about the transport function of the

human lymphatic system as investigated in vitro and in vivo. Due to lack of invstigational tools, the knowledge  of the function of the human lymphatic system has been limited so far. The possibility to investigate the lymphatic system clinically has opened new investigational avenues and has demonstrated that this overlooked system plays an important role in our fluid homeostasis. Despite being a low flow - slow flow system, recent publications incoorporated in the present systematic review have demonstrated a fluid transport system that reacts within minutes to daily occuring events such as temperature changes and positional changes.

Very recent animal studies have pointed towards an importance of this system in common and

life threathening diseases such as heart failure. Although the current literature on humans is not there yet,- a growing interest is present and we foresee that a systematic review about the function of the peripheral lymphatic system is a timely contribution to this emerging field.

Thank you for considering this manuscript. We look forward to hearing from you.

Sincerely,

Lene Thorup

MD, Ph.d.-student

Department of Cardiothoracic Surgery

Copenhagen University Hospital

Copenhagen, Denmark
